# Association of daily physical activity with pulmonary artery pressure in HFpEF and HFmrEF NYHA class III patients: a pilot trial—feasibility and first results

**DOI:** 10.1007/s00392-024-02564-6

**Published:** 2024-11-07

**Authors:** Ester J. Herrmann, Denise Lange, Jennifer Hannig, Gina Zimmer, Dimitri Gruen, Till Keller, Albin Edegran, Linda S. Johnson, Samuel Sossalla, Michael Guckert, Birgit Assmus

**Affiliations:** 1 Department of Medicine I, Cardiology and Angiology, University Hospital Giessen and Marburg, Justus Liebig University, Klinikstr. 33, 35392 Giessen, Germany; 2 Institute of Mathematics, Natural Sciences and Data Processing, Technische Hochschule Mittelhessen, University of Applied Sciences, 61169 Friedberg, Germany; 3 Cognitive Information Systems Group, Kompetenzzentrum Für Informationstechnologie (KITE), Technische Hochschule Mittelhessen, University of Applied Sciences, 61169 Friedberg, Germany; 4 Justus-Liebig-University, 35392 Giessen, Germany; 5 MEDICALgorithmics, Warsaw, Poland; 6 Department of Clinical Sciences, Lund University, Skåne University Hospital, Malmö, Sweden; 7 German Center for Cardiovascular Research (DZHK), Partner Site Rhein, Main, Germany; 8 Present Address: Justus-Liebig-University, 35392 Giessen, Germany

**Keywords:** Heart failure with preserved ejection fraction, Daily activity, Pulmonary artery pressure, MET score

## Abstract

**Introduction:**

Supervised physical exercise has been shown to benefit patients with heart failure with preserved/mildly reduced ejection fraction (HFpEF/HfmrEF) by improving symptoms and diastolic function. This study aimed to investigate the correlation between unsupervised daily physical activity and changes in daily pulmonary artery pressure (PAP) in patients with stable NYHA class III heart failure (HF) and left ventricular ejection fraction (LVEF) of 45% or higher.

**Methods:**

Daily physical activity was monitored over a 3-month period using a Holter-ECG with an accelerometer that calculated an activity-associated, heart rate-derived metabolic equivalent of task (MET) score. PAP was measured using an implanted sensor in 17 patients.

**Results:**

During 3 months of PAP monitoring in parallel with Holter ECG in our HF patients (median age 77 [IQR 72–79.5] years, LVEF 55 [49–56] %, mean cardiac index 1.9 ± 0.3), mean, diastolic, and systolic PAP remained unchanged. Patients engaged in unsupervised daily activity with a mean MET score of 5.0 ± 1.2 and a median daily duration of 41 [13–123] minutes. Intensity of daily activity was associated with a higher diastolic PAP on the following day (R^2^ = 0.017, p = 0.003), particularly in female patients and those with pulmonary hypertension (PH) (female: R^2^ = 0.044, p = 0.002; PH: R^2^ = 0.024, p = 0.004). Patients with longer daily activity durations had lower systolic and mean PAP (p = 0.038 and p = 0.048) and a similar diastolic PAP (p = 0.053) after 3 months.

**Conclusions:**

Tracking changes in daily PAP based on intensity and duration of unsupervised daily activity using implanted sensors and a PocketECG^®^ is feasible. While daily activity duration was not directly linked to diastolic PAP on the first day after daily activity, intensity, especially in female and PH patients, was associated with increased diastolic PAP. In addition, longer daily activity, rather than higher intensity, might be more important for lowering PAP in the long term. Further research in larger trials is warranted to confirm these findings.

**Supplementary Information:**

The online version contains supplementary material available at 10.1007/s00392-024-02564-6.

## Introduction

Heart failure (HF) with preserved ejection fraction (HFpEF) is currently the most common form of HF, and it is increasing in prevalence due to the ageing of the population [1, 2]. Further risk factors include arterial hypertension, previous myocardial infarction, obesity, and a sedentary lifestyle [3, 4]. Exercise intolerance associated with reduced quality of life (QOL) is a cardinal feature of HFpEF [5], and this is perpetuated by sedentary behavior, deconditioning, and frailty [6]. Verified therapeutic options improving exercise capacity are rare in HFpEF patients compared with those with HF with reduced ejection fraction (HFrEF). Recommended treatment approaches in HFpEF patients include SGLT2-inhibitors [7], and there are suggested beneficial effects of the GLP-1 agonist semaglutide in obese patients with HFpEF [8]. Other treatment options include exercise training [9] and weight loss [10].

Exercise intolerance can be measured as impaired peak oxygen consumed during maximal effort exercise (peak VO2). Reduced exercise capacity is independently associated with worse outcome [11]. The metabolic equivalent of task (MET) score is a universally used parameter that represents the energy cost of physical activity (VO2). A predicted MET value (peak VO2) can be derived using the heart rate index (HRI) equation, which is not statistically different from using the measured treadmill-derived peak VO2 [12].

In patients with HF, the presence of secondary pulmonary hypertension (PH) is associated with reduced exercise capacity and worse prognosis [13–16]. Previous data showed that a 5-mmHg reduction in the estimated diastolic pulmonary artery pressure (PAP) was associated with a 30% survival benefit after 6 months [17]. Thus far, it is not known whether unsupervised spontaneous daily activity has any impact on PAP or whether daily activity is associated with a decline or an increase in PAP in specific phenotypes. Therefore, the aim of this study was to investigate whether the duration or the intensity of unsupervised spontaneous daily activity has a direct impact on diastolic PAP assessed 12 to 24 h later.

## Methods

The present analysis is a prospective, open-label single-center registry including patients with an implanted pulmonary artery pressure sensor. In the present cohort, patients received also a 3 months Holter ECG for heart rate and activity assessment. However, all acquired data were not available for the patients.

### Cohort

Patients with chronic HF with preserved or mildly reduced ejection fraction (HFpEF/HFmrEF, LVEF 45%) in NYHA functional class III and a cardiac decompensation event within the last 12 months who were on individually optimized medical therapy were offered implantation of the PAP sensor (CardioMEMS™, Abbott, Sylmar, CA, USA) and participation in a center telemonitoring registry. Patients received PAP-guided HF management between 2020 and 2022 and were repeatedly trained in HF self-care by a European Society of Cardiology (ESC)-certified HF nurse.

All patients who were implanted with the PAP sensor (CardioMEMS™) provided written informed consent for participation in the center registry (NCT03020043). The study was approved by the local ethics committee and complied with the principles laid out in the Declaration of Helsinki.

### PocketECG^®^

HFpEF/HFmrEF patients were fitted with a PocketECG^®^ (MEDICALgorithmics S.A., Warsaw, Poland) immediately prior to discharge after implantation of the PAP sensor and were asked to wear it for three months. The PocketECG^®^ device is a full-disclosure ambulatory ECG device with a limb-lead configuration, that is equipped with an accelerometer that detects periods with and without physical activity and measures its intensity, classifying it into low, moderate and high intensity. This allows accurate calculation of the resting heart rate (HR). We defined resting as a period of time starting at least 10 min after moderate or intense activity, at least 5 min after low activity, or at least 30 s after the median of accelerometer energy crossed the low-level activity threshold but did not last long enough to be qualified as increased activity. Rest periods end when the acceleration crosses into low or higher activity.

The MET score was calculated for each patient according to the following formula [12]:

$${\mathrm{MET}}\, = \,{6} \times {\text{HR }}/{\text{ resting HR}} - {5}$$

Resting HR was calculated separately for each day of monitoring as a median HR during "rest". The MET score was then calculated separately for each beat based on current HR and daily resting HR, so that each heartbeat over the entire duration of the recording was assigned a MET score value.

The pulse pressure (PP) was calculated as the difference between systolic PAP and diastolic PAP, and the proportional PP was computed as the ratio of PP to systolic PAP.

In addition, the distance achieved in a 6-min walk and core laboratory determination of N-terminal fragment of pro-brain natriuretic peptide (NT-proBNP) levels were assessed at baseline and after 3 months.

### Data preprocessing

Data preprocessing steps were applied to eliminate artefacts and implausible constellations and to prepare data for the analysis steps. Days of ECG recordings with less than 80% of diagnostic ECG signals per day were excluded completely from the analysis. Nocturnal beats, negative values, beats without timestamp, beats without MET scores, and arrhythmic beats like couplets, triplets, and bigeminy (Supplementary Table 1) were removed as well as beats with an HR < 25 min^−1^ and > 150 min^−1^.

Activity data gained from ECG devices was originally measured in short pulses three seconds in length. Assuming that human activity will typically expand over longer periods, these measurements were aggregated into blocks with a length of one minute each during preprocessing. Therefore, the basic unit of the analyses is in minutes. This is in line with the well-known fact that one minute of activity can be sufficient to double PAP [18]. The intensity of a given minute is then computed as the mean of all short MET score measurements that fall within that minute.

Depending on the computed average of intensity, a minute is either classified as active (MET score ≥ 3) or at rest (MET score < 3). Thereafter, consecutive minutes of the same type (activity or rest) were aggregated into periods of either activity or rest. More precisely, for two given minutes T_1_ and T_2_ with T_1_ < T_2_, both either active or at rest and no T’ with T_1_ < T’ < T_2_ with a different classification, we get a period T (starting at T_1_, ending at T_2_) of activity or rest of length.

$${\mathrm{T}}\, = \,{\mathrm{T}}_{{2}} {-}{\mathrm{T}}_{{1}}$$

Activity data and rest data were examined separately. For examination of daily intensity and duration of activity, the activity period data was aggregated on a daily basis by calculating the sum of activity durations and the mean of activity intensity for each day.

Changes in PAP were examined the day after exercise. Reference data was obtained by calculating the mean PAP in a neighborhood of the day observed. The neighborhood consisted of the observed day, the following day, and up to three preceding days, depending on availability. Reference data was compared with the absolute PAP of the day following the observed day.

Additionally, the mean of daily intensity and duration of activity data for the whole study period was calculated for each patient. The cohort was then dichotomized at the median values for duration and intensity of daily activity into lower and upper portions. For both portions, the change in PAP between 3 days post-discharge and the 3-month follow-up was compared for the respective groups.

### Statistical analysis

Baseline characteristics were calculated either as median and interquartile range (IQR) or mean and standard deviation (SD) (the Shapiro–Wilk test was applied for confirming normal distribution). Student’s t-test was used to compare 3-day post-discharge with 3-month follow-up values (PAP, PP, proportional PP), whereas 6-min walking distance and NT-ProBNP levels were compared between baseline and the 3-month follow-up.

Linear mixed-effects regression was applied to analyze a potential association between duration and intensity of daily activity with diastolic PAP the first day after daily activity.

The analysis considered changes within or between the corresponding groups for lower and upper PAP using Student’s t-test. Statistical significance was assumed if p < 0.05, and all reported p-values are two-sided.

Statistical analysis was carried out with the software packages SPSS (Version 29.0.1.1, SPSS Inc., IBM Corp., Armonk, NY, USA) and in Python (Version 3.8.13, Python Software Foundation, Delaware, USA) and the packages numpy (Version 1.23.1), pandas (Version 1.4.3), matplotlib (Version 3.5.2) and statsmodels (Version 0.13.2).

## Results

### Baseline characteristics

**Fig. 1:**
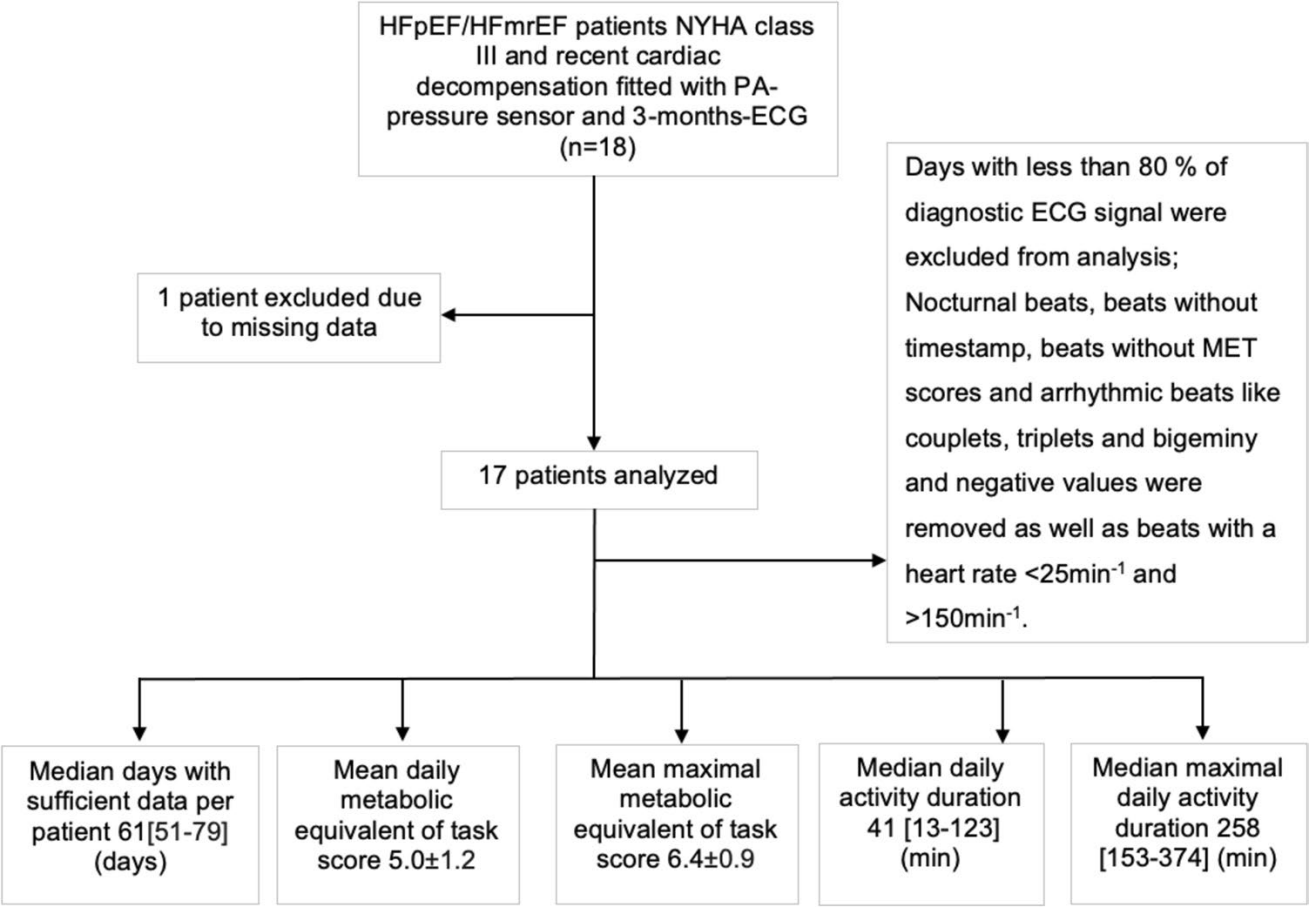
Study Flowchart. HFpEF, heart failure with preserved ejection fraction; HFmrEF, heart failure with mildly reduced ejection fraction; ECG, electrocardiogram; PA, pulmonary artery; NYHA, New York Heart Association

**Fig. 2:**
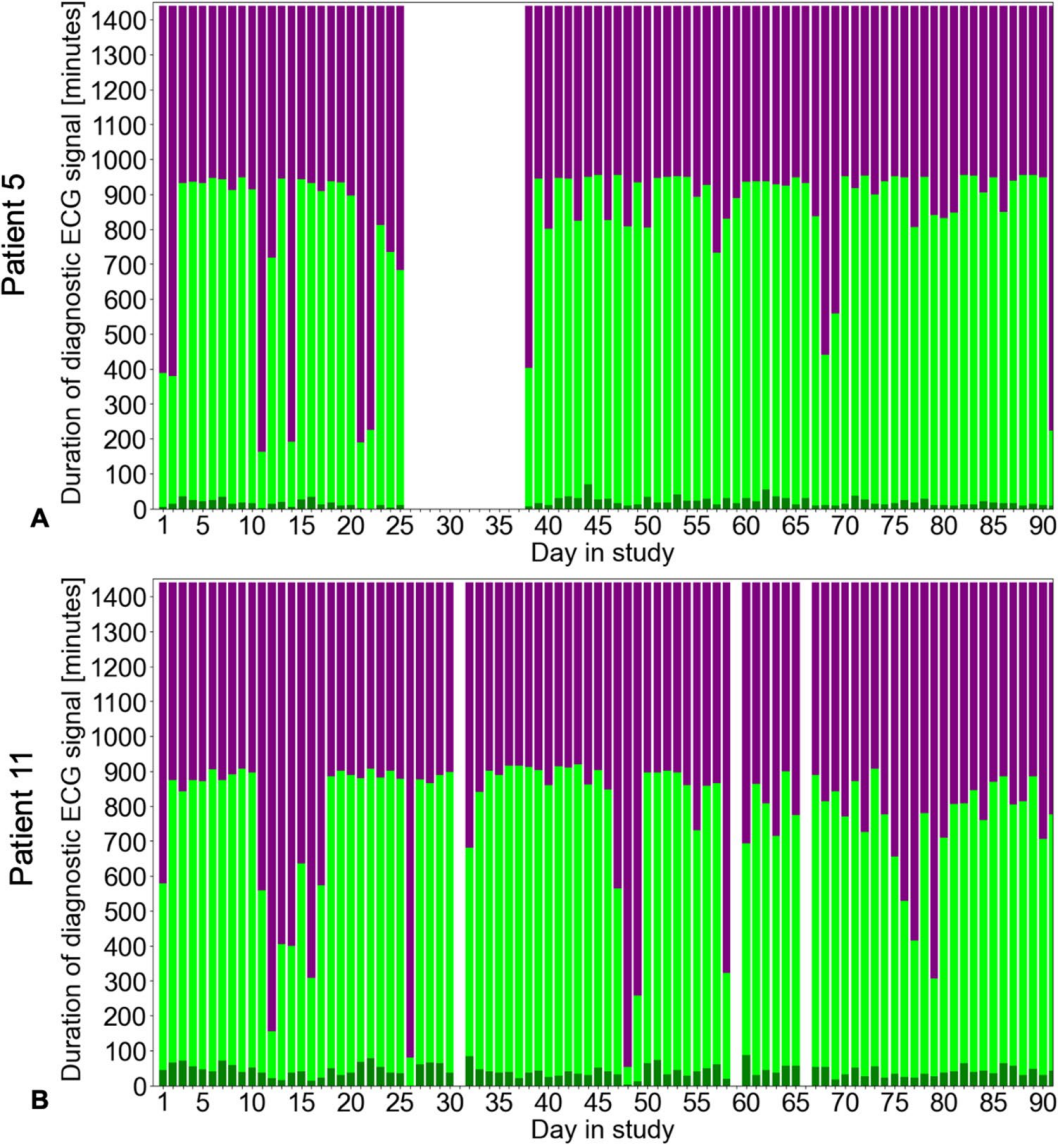
Data coverage of diagnostic ECG in minutes during the study. Examples show data from a patient (# 5) with sinus rhythm and without pulmonary hypertension (**A**), and from a patient (# 11) with pulmonary hypertension (**B**). Dark green, duration of activity; light green, duration of rest; purple, missing duration. ECG, electrocardiogram

Of 18 patients fitted with a PAP sensor and 3-month PocketECG^®^, one patient was excluded because ECG tracking was possible for only 10 days due to an allergic skin reaction to ECG electrodes, and only four PAP measurements could be obtained due to attacks of gout. Therefore, 17 patients were analyzed in our study, wearing the PocketECG^®^ for a total of 80 ± 14 days. From these data, we obtained 61 ± 21 days (mean ± SD) per patient with sufficient ECG data, a MET score of 5.0 ± 1.2, a maximal MET score of 6.4 ± 0.9, a median duration of daily activity of 41 [13–123] minutes, and a median maximal duration of 258 [153–374] minutes (Fig. 1). Using filter criteria, 19% of beats and 9% of days were excluded (Supplementary Table 2). The data coverage over the days of the study period is shown exemplarily for two patients in sinus rhythm, one without (Patient #5; Fig. 2A) and one with (Patient #11; Fig. 2B) pulmonary hypertension (PH).

Our cohort consisted of 17 patients with NYHA class III chronic HF. The median age was 77 [72–79.5] years, 52.9% were female, 88.2% had HFpEF and 11.8% had HFmrEF. About one-quarter of patients (23.5%) suffered from diabetes mellitus type 2, 70.6% from atrial fibrillation, and 94.1% from arterial hypertension (Table 1). The median LVEF was 55 [49–56] % and the cardiac index was 1.9 ± 0.3. Fourteen of 17 patients (82.4%) had PH (PAPmean > 20 mmHg), and of those, 5 (29.4%) presented with a combined pre-and post-capillary PH phenotype according to the 2022 ESC Guidelines [14] with a PVR > 2 WU (Table 2).

**Table 1:** Baseline characteristics

	Study cohort (n = 17)
Age, years	77 (72–79.5)
Sex	
Male	8 (47.1%)
Female	9 (52.9%)
BMI, kg/m^2^	30 ± 4
NYHA functional class	
III	17 (100%)
Medical history	
Previous myocardial infarction	1 (5.9%)
Previous percutaneous coronary intervention	1 (5.9%)
Previous coronary artery bypass grafting	1 (5.9%)
Diabetes mellitus type 2	4 (23.5%)
Cerebrovascular accident or transient ischemic attack	1 (5.9%)
Atrial fibrillation	12 (70.6%)
Arterial hypertension	16 (94.1%)
Years since heart failure diagnosis	3 (1.3–5)
Cause	
Ischemic	1 (5.9%)
Heart rate, beats per min	70 ± 12
Systolic blood pressure, mmHg	128 ± 18
6-min walking test, m	298 ± 56
Type of heart failure	
HFpEF	15 (88.2%)
HFmrEF	2 (11.8%)
HFrEF	0 (0%)
Serum creatinine, mg/dl	1.35 ± 0.43
eGFR, ml/min	52 ± 16
Chronic kidney disease	
KDIGO Grade 1	6 (35.3%)
KDIGO Grade 2	2 (11.8%)
KDIGO Grade 3	8 (47.1%)
KDIGO Grade 4	1 (5.9%)
KDIGO Grade 5	0 (0%)
NT-proBNP, pg/ml	1388 ± 1266
Implanted cardioverter defibrillator	2 (11.8%)
Cardiac resynchronization therapy	2 (11.8%)
Medical therapy	
Beta-blocker	13 (76.5%)
Renin angiotensin system inhibitor	8 (47%)
Angiotensin converting enzyme inhibitor	4 (23.5%)
Angiotensin receptor blocker	4 (23.5%)
Angiotensin-receptor neprilysin inhibitor	4 (23.5%)
Mineralocorticoid receptor antagonist	10 (58.8%)
SGLT-2 inhibitor	2 (11.8%)
Loop diuretic	15 (88.2%)
Loop diuretic torasemide dose equivalent, mg	15 (10–30)
Thiazide diuretic	4 (23.5%)
Combined loop and thiazide diuretic	4 (23.5%)

All data are presented in n (%), median (IQR) or mean ± SD

*HFpEF* heart failure with preserved ejection fraction, *HFmrEF* heart failure with mildly reduced ejection fraction, *HFrEF* heart failure with reduced ejection fraction, *eGFR* estimated glomerular filtration rate, *KDIGO* Kidney Disease Improving Global Outcomes, *NT-proBNP* N-terminal fragment of pro-brain natriuretic peptide, *SGLT-2 inhibitor* sodium glucose-linked transporter-2 inhibitor

**Table 2:** Echocardiography and invasive hemodynamics at baseline

	Study cohort (n = 17)
Echocardiography at baseline	
Quantitative left ventricular ejection fraction (%)	55 (49–56)
TAPSE (mm)	23.5 (17–26)
Right ventricular fractional area change (%)	40 ± 8
TAPSE/PASP ratio, (echo, mm/mm Hg)	0.54 ± 0.23
TAPSE/PASP ratio, (CardioMEMS™, mm/mmHg)	0.49 ± 0.19
Invasive hemodynamics at index procedure	
Cardiac index (l/min/m^2^)	1.9 ± 0.3
Systolic PAP (mmHg)	45 ± 12
Diastolic PAP (mmHg)	16 ± 8
Mean PAP (mmHg)	28 ± 8
Pulmonary capillary wedge pressure (mmHg)	20 ± 5
Pulse Pressure (Systolic-diastolic PAP, mmHg)	29 ± 10
Proportional pulse pressure (PP/systolic PAP)	0.64 ± 0.13
Pulmonary vascular resistance (Wood units)	2.0 (1.5–3.1)
Pulmonary hypertension	
None	3 (17.6%)
Isolated postcapillary pulmonary hypertension	14 (82.4%)
Combined pre- and postcapillary pulmonary hypertension	5 (29.4%)

All data are presented in n (%), mean ± standard deviation or median (IQR). TAPSE, tricuspid annular plane systolic excursion. PASP, systolic pulmonary artery pressure

All patients received guideline-recommended treatment for comorbidities. Of note, SGLT-2 inhibitors were not yet recommended by guidelines during the recruitment period.

### Follow-up parameters

During 3 months of hemodynamic monitoring in parallel with PocketECG^®^ monitoring, the diastolic, mean, and systolic pressures remained stable with the application of HF-guided care (absolute changes in diastolic PAP: 0 ± 6 mmHg, p = 0.972; mean PAP: 0 ± 8 mmHg, p = 0.74; systolic PAP 0 ± 9 mmHg, p = 0.512) (Table 3). At the 3-month follow-up, neither the median 6-min walking distance (baseline 289 ± 56 m, 3 months 309 ± 53 m) nor the NT-proBNP levels (baseline 1388 ± 1266 pg/ml, 3 months 1430 ± 1160 pg/ml) were significantly different from baseline (Table 3).

**Table 3:** Pulmonary artery pressure at 3 days post-discharge versus at 3-month follow-up as well as exercise capacity and NT-proBNP

	Study cohort (n = 17)		
PAP sensor-derived values at 3 days post-discharge			
Systolic PAP (mmHg)	48 ± 9		
Diastolic PAP (mmHg)	23 ± 6		
Mean PAP (mmHg)	33 ± 7		
Pulse pressure (mmHg)	25 ± 6		
Proportional pulse pressure	0.53 ± 0.09		
PAP sensor-derived values at 3-month follow-up			
Systolic PAP (mmHg)	47 ± 14		
Diastolic PAP (mmHg)	23 ± 7		
Mean PAP (mmHg)	33 ± 10		
Pulse pressure (mmHg)	24 ± 9		
Proportional pulse pressure	0.51 ± 0.10		
Absolute changes from 3 days post-discharge to 3-month follow-up			
Systolic PAP (mmHg)	0 ± 9, p = 0.907		
Diastolic PAP (mmHg)	0 ± 6, p = 0.838		
Mean PAP (mmHg)	0 ± 8, p = 0.904		
Pulse pressure (mmHg)	-1 ± 5, p = 0.724		
Proportional pulse pressure	-0.02 ± 0.08, p = 0.629		
Absolute changes from 3 days post-discharge to 3-month follow-up dichotomized by median of duration of daily activity			
Changes in upper subcohort (duration > 48 min)		Intergroup	Intragroup
Systolic PAP (mmHg)	-5 ± 7	p* = 0.038	p^#^ = 0.210
Diastolic PAP (mmHg)	-3 ± 7	p* = 0.053	p^#^ = 0.269
Mean PAP (mmHg)	-4 ± 7	p* = 0.048	p^#^ = 0.246
Pulse pressure (mmHg)	-3 ± 5	p* = 0.206	p^#^ = 0.424
Proportional pulse pressure	0 ± 0.11	p* = 0.532	p^#^ = 0.954
Changes in lower subcohort (duration ≤ 48 min)			
Systolic PAP (mmHg)	4 ± 9		p^#^ = 0.575
Diastolic PAP (mmHg)	3 ± 5		p^#^ = 0.403
Mean PAP (mmHg)	4 ± 7		p^#^ = 0.448
Pulse pressure (mmHg)	1 ± 5		p^#^ = 0.881
Proportional pulse pressure	-0.03 ± 0.05		p^#^ = 0.464
Absolute changes from 3 days post-discharge to 3-month follow-up dichotomized by median of intensity of daily activity			
Changes in upper subcohort (intensity > 5 MET)		Intergroup	Intragroup
Systolic PAP (mmHg)	-3 ± 5	p* = 0.251	p^#^ = 0.450
Diastolic PAP (mmHg)	0 ± 6	p* = 0.619	p^#^ = 0.904
Mean PAP (mmHg)	-1 ± 6	p* = 0.437	p^#^ = 0.718
Pulse pressure (mmHg)	-3 ± 4	p* = 0.143	p^#^ = 0.361
Proportional pulse pressure	-0.03 ± 0.10	p* = 0.588	p^#^ = 0.609
Changes in lower subcohort (intensity ≤ 5 MET)			
Systolic PAP (mmHg)	2 ± 11		p^#^ = 0.762
Diastolic PAP (mmHg)	1 ± 7		p^#^ = 0.733
Mean PAP (mmHg)	2 ± 9		p^#^ = 0.706
Pulse pressure (mmHg)	1 ± 6		p^#^ = 0.847
Proportional pulse pressure	-0.01 ± 0.07		p^#^ = 0.892
6-min walking test at 3 months (m)	309 ± 53		
Absolute change in 6-min walking test at 3-month follow-up (m)	15 ± 25, p = 0.574		
NT-proBNP level at 3 months follow-up (pg/ml)	1430 ± 1160		
Absolute change in NT-proBNP value at 3-month follow-up (pg/ml)	130 ± 656, p = 0.993		

Values represent mean ± standard deviation; p-values are two-sided

*PAP* pulmonary artery pressure, *MET* metabolic equivalent of task score, *NT-proBNP* N-terminal fragment of pro-brain natriuretic peptide

p*-value represents comparison of PAP between patients below and above the median values of the duration or the intensity of daily activity

p^#^-value represents intraindividual change measured at 3 days vs. at the 3-month follow-up

Overall, there was no significant correlation found between changes in PAP and unsupervised daily activity levels at the 3-months follow-up. However, the 8 patients with a mean daily activity duration above the median value (> 48 min) had a significantly lower systolic and mean PAP (p = 0.038 and p = 0.048, respectively), with no significant difference in diastolic PAP (p = 0.053), compared with the 9 patients with a mean activity duration below the median value of ≤ 48 min (Table 3). On the other hand, there was no significant difference in PAP between patients with a higher intensity of daily activity (MET > 5) and those with a lower intensity (MET ≤ 5) after 3 months.

### Duration and intensity of daily activity versus diastolic PAP in two representative patients

**Fig. 3:**
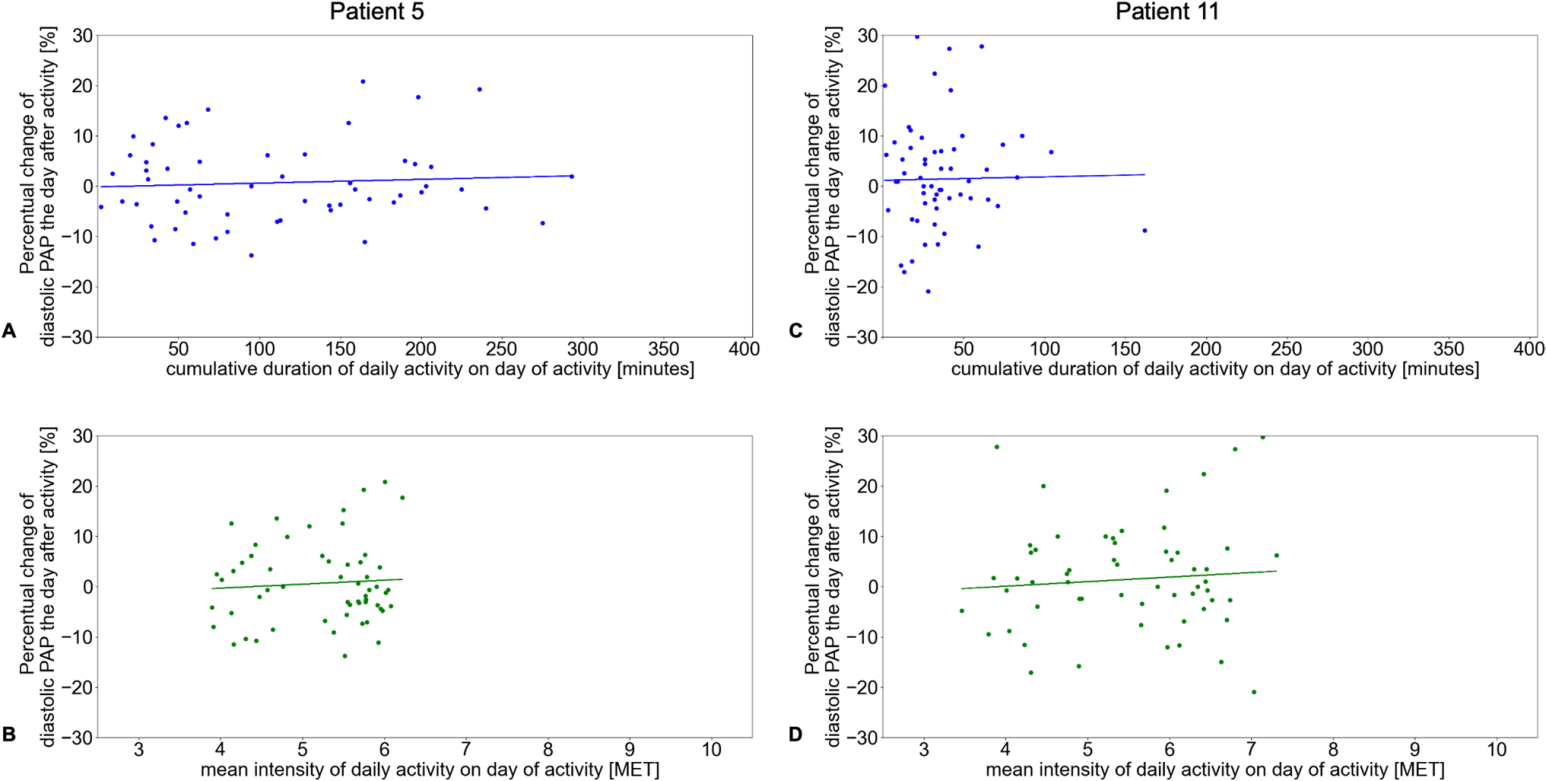
Correlation of daily activity and duration versus relative change in diastolic PAP on day one after the corresponding activity. The reference PAP is the mean of the observed day, the following day, and up to three preceding days, depending on availability. Examples show data from a patient (# 5) with sinus rhythm and without pulmonary hypertension (**A**, **B**), and from a patient (# 11) with pulmonary hypertension (**C**, **D**). PAP, pulmonary artery pressure. MET, metabolic equivalent of task

**Fig. 4:**
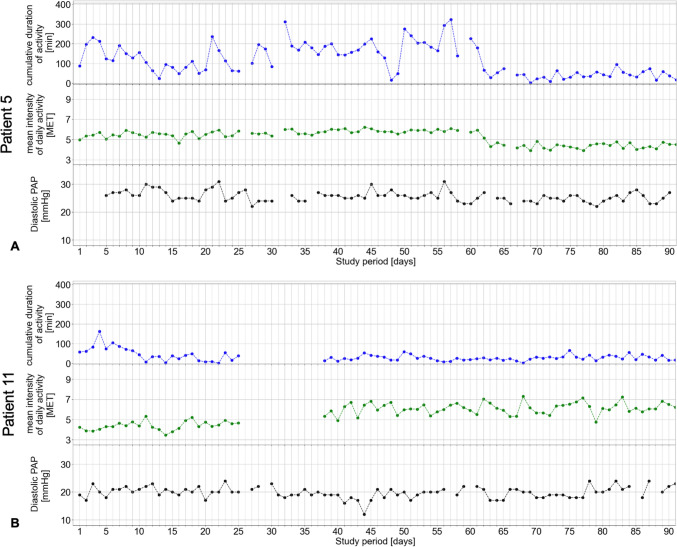
Association of diastolic PAP and intensity and duration of daily activity over the study period. **A** Patient 5 without pulmonary hypertension. **B** Patient 11 with pulmonary hypertension. Blue, Duration of daily activity. Green, intensity of daily activity. Black, morning diastolic PAP over the study period. MET, metabolic equivalent of task

**Fig. 5:**
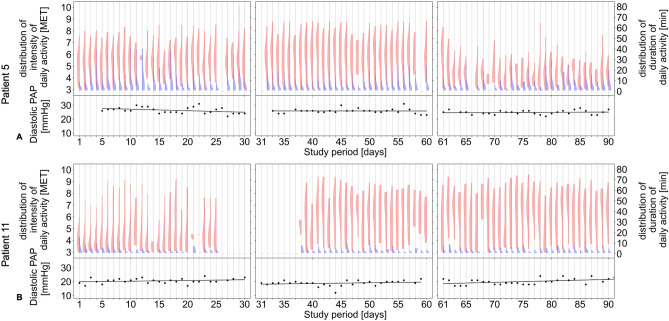
Split violin plots of intensity and duration of daily activity in parallel with diastolic pulmonary artery pressure for the 3 months in the follow-up period. Red split violin, intensity of daily activity. Blue split violin, duration of daily activity. Each period of duration of daily activity is plotted individually. MET, metabolic equivalent of task

The relative change in diastolic PAP one day after daily activity plotted as a function of the duration and intensity of daily activity is presented for two patients in sinus rhythm, Patient #5 without (Fig. 3A and B) and Patient #11 with (Fig. 3C and D) PH. For these two representative patients, the intensity and duration of daily activity as well as diastolic PAP recorded over the entire duration of the study period is shown in Fig. 4A and B. For better visualization of the distribution, we plotted the duration and intensity of daily activity via split violin plots in parallel with the corresponding diastolic PAP for the abovementioned patients without (Fig. 5A) and with (Fig. 5B) PH.

### Association of duration and intensity of daily activity with diastolic PAP

To assess the immediate impact of unsupervised exercise on subsequent PA pressure, we analyzed the impact of activity intensity and duration on each day on PA pressures on the following day. Although there was no association for the entire cohort between the duration of daily activity and diastolic PAP measured one day after the activity (R^2^ = 0.005, p = 0.100), there was a significant association of the intensity of daily activity and subsequent diastolic PAP (R^2^ = 0.017, p = 0.003).

**Fig. 6:**
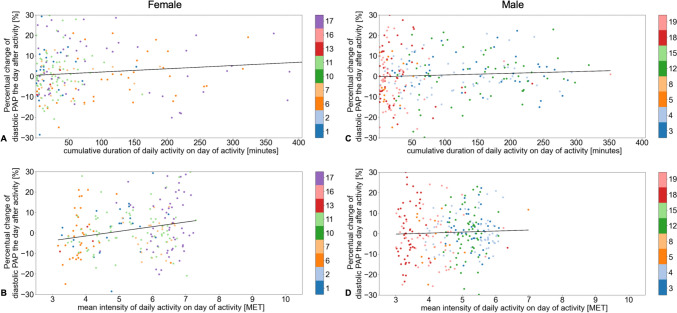
Correlation between duration or intensity of daily activity for female or male patients and change in diastolic pulmonary artery pressure one day after daily activity. The reference pulmonary artery pressure (PAP) was calculated as the mean of the observed day, the following day, and up to three preceding days, depending on availability. The percentual changes in diastolic PAP according to duration of daily activity for female (**A**) and male (**C**) patients, and according to intensity of daily activity (metabolic equivalent of task [MET] score) for female (**B**) and male (**D**) patients are shown

**Fig. 7:**
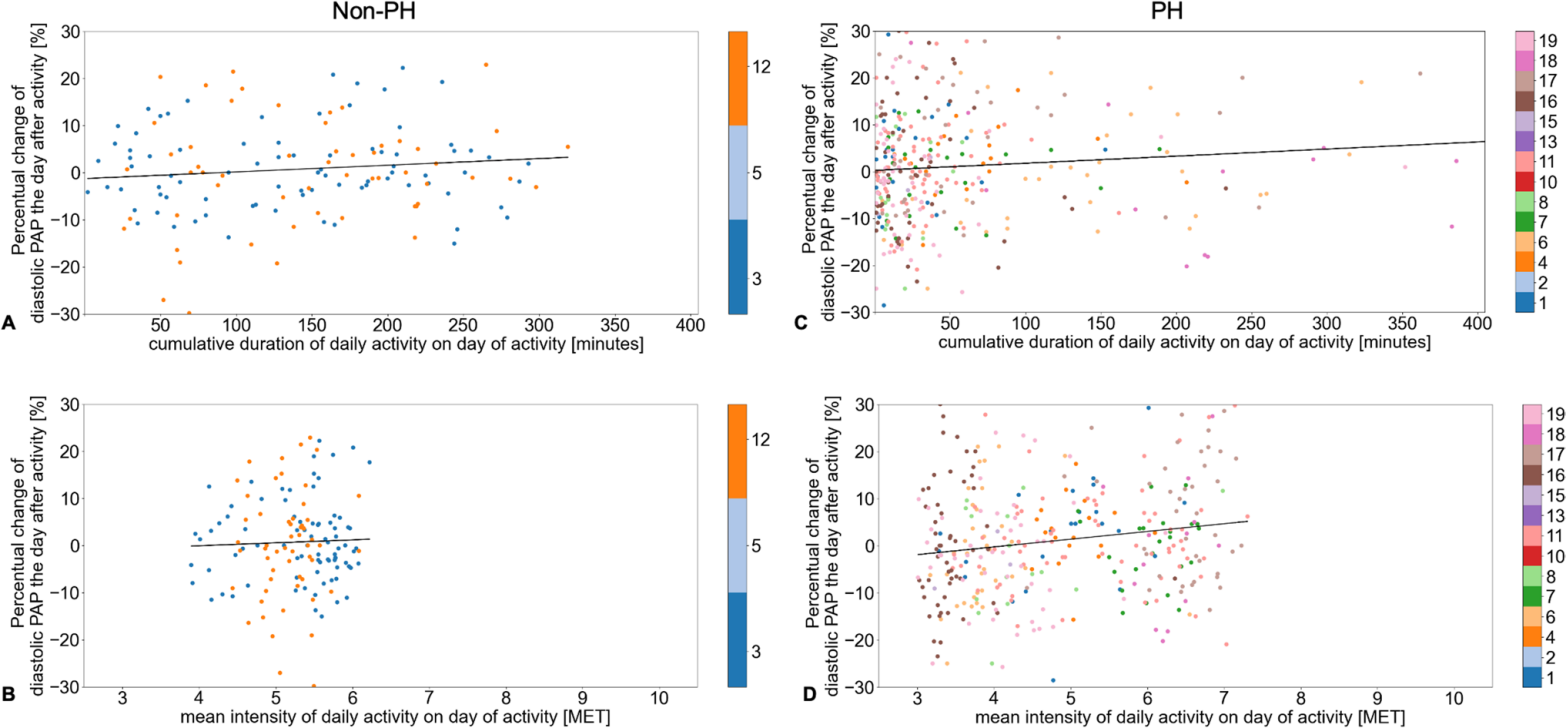
Correlation between duration or intensity of daily activity for patients with or without pulmonary hypertension and change in diastolic pulmonary artery pressure one day after daily activity. The reference pulmonary artery pressure (PAP) was calculated as the mean of the observed day, the following day, and up to three preceding days, depending on availability. The percentual changes in diastolic PAP according to duration of daily activity for patients without pulmonary hypertension (PH) (**A**), with PH (**C**), and according to intensity of daily activity (metabolic equivalent of task [MET] score) for patients without PH (**B**) and with PH (**D**) are shown

Subgroup analysis of patients with or without underlying PH, or of male or female patients, did not reveal an association of daily activity duration (PH: R^2^ = 0.007, p = 0.105; non-PH: R^2^ = 0.011, p = 0.221; male: R^2^ = 0.003, p = 0.335; female: R^2^ = 0.011, p = 0.125) or intensity with diastolic PAP the day after activity in non-PH and male patients (non-PH: R^2^ = 0.001, p = 0.720; male: R^2^ = 0.001, p = 0.536), but the intensity of daily activity did correlate with diastolic PAP in female and PH patients (female: R^2^ = 0.044, p = 0.002; PH: R^2^ = 0.024, p = 0.004) (Figs. 6 and 7). Patients in sinus rhythm presented with a higher intensity of daily activity and had higher diastolic PAP values the first day after activity (R^2^ = 0.025, p = 0.010), whereas the duration of daily activity in these patients did not correlate with diastolic PAP (R^2^ = 0.002, p = 0.456). In patients with atrial fibrillation, there was no association between the duration (R^2^ = 0.007, p = 0.195) or intensity (R^2^ = 0.005, p = 0.291) of daily activity with diastolic PAP (Supplementary Fig. 1).

## Discussion

To the best of our knowledge, the current analysis of this case series represents the first prospective investigation of the level of unsupervised daily activity and daily assessed PAP in a representative HFpEF/HFmrEF patient cohort of NYHA class III managed by non-invasive PAP-guided HF care. Our findings indicate that measurement of PAP changes in relation to the intensity and duration of daily activity using PAP-guided monitoring combined with PocketECG^®^ is feasible. The technique represents a novel approach to investigating the relation of daily activity and PAP changes. This is in line with the findings of a small observational study from Mullens et al. with 12 HF patients using the Cordella™ PA Sensor system for continuous, non-invasive, untethered PAP measurement during 6-min walking test [19].

The duration of daily activity was not associated with the diastolic PAP assessed on the first day after the activity, but a higher intensity of daily activity was associated with a higher subsequent diastolic PAP in all patients as well as in female and PH patients (Fig. 6B and 7D). Because 82.4% of our cohort suffered from PH, this might be one of the leading contributing factors, but the total cohort as well as the non-PH cohort were far too small for any comprehensive subgroup analyses. As secondary PH is common in HFpEF patients and is associated with reduced exercise capacity and worse prognosis [13–16], a higher diastolic PAP after activity and even worsening PAP with increasing intensity of daily activity could therefore explain at least in part the patients’ reluctance to participate in exercise training. However, our data also suggest that a longer duration rather than a higher intensity of daily activity might be more important for a decrease of PAP even in PH patients in the long-term (Table 3). Our findings underline the fact that HFpEF patients with PH differ in hemodynamics and exercise capacity from HFpEF patients without PH [20], although we only examined the effect of unsupervised daily activity. PH training studies usually exclude patients with post-capillary PH, and HFpEF exercise trials do not focus on pulmonary circulation [21]. The currently ongoing TRAIN-HFpEF-PH trial investigates whether a standardized exercise training program is safe and tolerable and may improve exercise capacity, quality of life, hemodynamics, diastolic dysfunction, and biomarkers in patients with HFpEF-PH [22]. Results are expected by the end of 2024.

It is known that physical activity assessed with an accelerometer decreases with age, which is independent of muscle mass [23], that it decreases with increasing severity of obesity [24, 25], and that it is lower in females [26]. Women with HFpEF suffer from greater exercise limitation and a lower quality of life than men, despite having a better survival [27]. In a retrospective study of 114 women and 47 men with HFpEF undergoing invasive rest and exercise hemodynamic testing, Beale et al. reported a smaller increase in stroke volume index and a greater increase in PCWP during exercise in women compared to men [28]. This is in line with the findings of our small cohort demonstrating that a higher intensity of daily activity but not a longer duration was associated with higher diastolic PAP the day after activity in female HFpEF patients (Fig. 6A and B).

The PocketECG^®^ device is a Holter ECG monitor equipped with an accelerometer for classifying daily activity, and MET scores describing the intensity of activity are calculated by utilizing the HRI equation. This method can result in underprediction of VO2max, especially in highly fit individuals, as reported by Kang et al. in 11,257 participants with an average MET score of 13.4 [29]. However, among the 60 studies used to develop the HRI equation, 38 included patients with chronic diseases such as coronary heart disease, congestive HF, diabetes, and pulmonary disorders [30]. The prediction agreement of Kang et al. shown at 40% of VO2max is encouraging, as this intensity (MET score ~ 5) represents a level of exertion often experienced in leisurely physical activities [31]. However, there was a significant overestimation by ~ 0.5 MET units when exercise was performed at 60 and 80% of VO2max [29]. It is quite likely that most of the VO2max values Kang et al. obtained were beyond the highest VO2 used for establishing the equation, and this could result in error in predictions. In our present case series, we investigated daily activity in NYHA class III HFpEF/HFmrEF patients without any training program, so that the main intensity was within the range of 40% of V02max where a strong correlation of VO2 calculated by the HRI equation and VO2 measured during exercise exists [29].

Atrial fibrillation is associated with a reduction in cardiac output, and HF and atrial fibrillation have a synergistic, negative impact on quality of life, exercise capacity, and repeat hospitalizations [32]. Unfortunately, the accelerometer technique and MET score calculation used by the PocketECG^®^ device are not validated for atrial fibrillation. However, an analysis of MET score in patients with and without atrial fibrillation was performed by Johnson V. et al. (Abstract presented at Heart Rhythm 2021). To exclude artefacts, we filtered our data by including only days with > 80% coverage of diagnostic ECG, knowing that atrial fibrillation often leads to errant ECG signals, negative MET score values, and arrhythmic beats, and by excluding beats with a heart rate < 25 min^−1^ and > 150 min^−1^ as recommended by the manufacturer. Nevertheless, we only found an association of the intensity of daily activity on diastolic PAP in patients with sinus rhythm but not in patients with atrial fibrillation (Supplementary Fig. 1). Thus, further investigations are needed to explore these parameters in patients with atrial fibrillation.

In a small study of 11 HFpEF patients, Fujimoto et al. showed that endurance training failed to impart favorable effects on cardiovascular stiffness and RV-PA coupling and function in HFpEF patients [33]. Of note, only 7 of 11 HFpEF patients completed the one-year training program. Our study did not investigate a training program but just normal daily activity, which was shown to be associated with a lower risk of HF-related hospital admissions and mortality and other health-related outcomes in chronic HF patients ^34,35^. Nevertheless, it is of profound interest whether it can be demonstrated, using the combination of PA sensor-guided HF-care and PocketECG^®^, that a structured training program in HF patients has an impact on diastolic PAP.

### Limitations

In keeping with this study being an analysis of a case series, the small patient cohort is a limitation. Thus, our conclusions from daily activity and not explicitly physical training can only be hypothesis generating. It should also be mentioned that the effects of daily activity on diastolic PAP could have been influenced particularly by diuretics and, although less frequently, by guideline-directed medical treatment adjustments that were implemented by the HF monitoring team in cases with increasing PAP (e.g. during the 3-month follow-up period, the dosage of diuretics was adjusted 33 times and the guideline-directed dosage was adjusted 19 times, both with up and down titrations). In addition, the PocketECG^®^ and heart rate-derived MET score calculation have not yet been validated in patients with atrial fibrillation, although the use of the filter criteria should have enhanced the data quality as a basis for our analysis. Finally, the subgroup size, especially of non-PH patients of which there were only 3, was very small. Thus, the possible differences between non-PH and PH patients must be investigated in larger trials.

## Conclusion

The detection of subtle PAP changes and their relationship to the intensity and duration of spontaneous daily activity is feasible through the use of an implanted PAP sensor in combination with an accelerometer-containing Holter ECG. Whereas the duration of daily activity did not correlate with diastolic PAP measured on the first day after the activity, a higher intensity of activity, especially in female and PH patients, was associated with minor increases in diastolic PAP. In addition, our data suggest that a longer duration rather than a higher intensity of daily activity might be more important for lowering PAP in the long term. Further investigation into the impact of unsupervised exercise on pulmonary hemodynamics in HFpEF/HFmrEF patients with PH will be needed, and it would be important to determine whether a PAP-guided, structured exercise program can lead to a decline in pulmonary pressure, which should be associated with improved outcomes and quality of life.

## Supplementary Information

Below is the link to the electronic supplementary material.

Supplementary file1 (DOCX 13 KB)

Supplementary file2 (DOCX 15 KB)

## Data Availability

The data are combined together and analyzed within the center registry (NCT03020043) and can be obtained by justified written request.
