# Supplementary material for: Association of daily physical activity with pulmonary artery pressure in HFpEF and HFmrEF NYHA class III patients: a pilot trial—feasibility and first results
Source: Clin Res Cardiol. 2024 Nov 7;115(3):435–48. doi: 10.1007/s00392-024-02564-6 (PMC12894114; doi:10.1007/s00392-024-02564-6)
Supplement: Supplementary file 1 — Supplementary file1 (DOCX 13 KB) [file 392_2024_2564_MOESM1_ESM.docx]

**Supplementary Figure 1: Correlation between duration or intensity of daily activity for patients with sinus rhythm or atrial fibrillation and change in diastolic pulmonary artery pressure one day after daily activity.** The reference pulmonary artery pressure (PAP) was calculated as the mean of the observed day, the following day, and up to three preceding days, depending on availability. The percentual changes in diastolic PAP according to duration of daily activity for patients in sinus rhythm (**A**) and for those with atrial fibrillation (**C**), and according to intensity of daily activity (metabolic equivalent of task [MET] score) for sinus rhythm (**B**) and atrial fibrillation (**D**) patients are shown.
