# Supplementary material for: Association of daily physical activity with pulmonary artery pressure in HFpEF and HFmrEF NYHA class III patients: a pilot trial—feasibility and first results
Source: Clin Res Cardiol. 2024 Nov 7;115(3):435–48. doi: 10.1007/s00392-024-02564-6 (PMC12894114; doi:10.1007/s00392-024-02564-6)
Supplement: Supplementary file 2 — Supplementary file2 (DOCX 15 KB) [file 392_2024_2564_MOESM2_ESM.docx]

**Supplementary Table 1: Excluded arrhythmic beats recorded by PocketECG**^®^

| **Excluded arrhythmic beats** |
| --- |
| Asystole |
| Missed Beat |
| Pause |
| 2nd Degree Atrioventricular Block |
| 3rd Degree Atrioventricular Block |
| Junctional Rhythm |
| Ventricular Ectopy |
| Ventricular Couplet |
| Ventricular Triplet |
| Ventricular Bigeminy |
| Ventricular Trigeminy |
| Idioventricular Rhythm |
| Accelerated Idioventricular Rhythm |
| Ventricular Tachycardia |
| Supraventricular Ectopy |
| Supraventricular Couplet |
| Supraventricular Triplet |
| Supraventricular Tachycardia |
| Atrial Bigeminy |
| Atrial Trigeminy |

**Supplementary Table 2:** Absolute and percentage of excluded beats and days using filter criteria to exclude non-diagnostic ECG

| **Filter criteria** | **Absolute number of beats removed** | **Percentage of beats removed** | **Absolute number of days removed** | **Percentage of days removed** |
| --- | --- | --- | --- | --- |
| **Excluding heart**  **rate <25 /min-^1^ and >150 / min-^1^** | 1,250,171 | 1.62 | 0 | 0 |
| **Excluding arrhythmic beats** | 176,554 | 0.23 | 0 | 0 |
| **Excluding beats without MET values** | 7,677 | 0.01 | 0 | 0 |
| **Excluding beats without timestamp** | 0 | 0 | 0 | 0 |
| **Excluding nightly measurements (between 10 pm and 4 am)** | 3,885,567 | 5.13 | 1 | 0.07 |
| **Excluding beats with negative MET values** | 8,666,655 | 12.07 | 60 | 4.44 |
| **Excluding days with >20% unknown ECG** | 1,032,103 | 1.64 | 60 | 4.63 |
| **Total excluded** | 15,018,727 | 19.48 | 117 | 8.65 |
